# Supplementary material for: LDHA-mediated ROS generation in chondrocytes is a potential therapeutic target for osteoarthritis
Source: Nat Commun. 2020 Jul 9;11:3427. doi: 10.1038/s41467-020-17242-0 (PMC7347613; doi:10.1038/s41467-020-17242-0)
Supplement: Supplementary file 1 — Supplementary Information [file 41467_2020_17242_MOESM1_ESM.pdf]

Supplementary Information

LDHA-mediated ROS generation in chondrocytes is a potential therapeutic target for osteoarthritis. Arra et al.

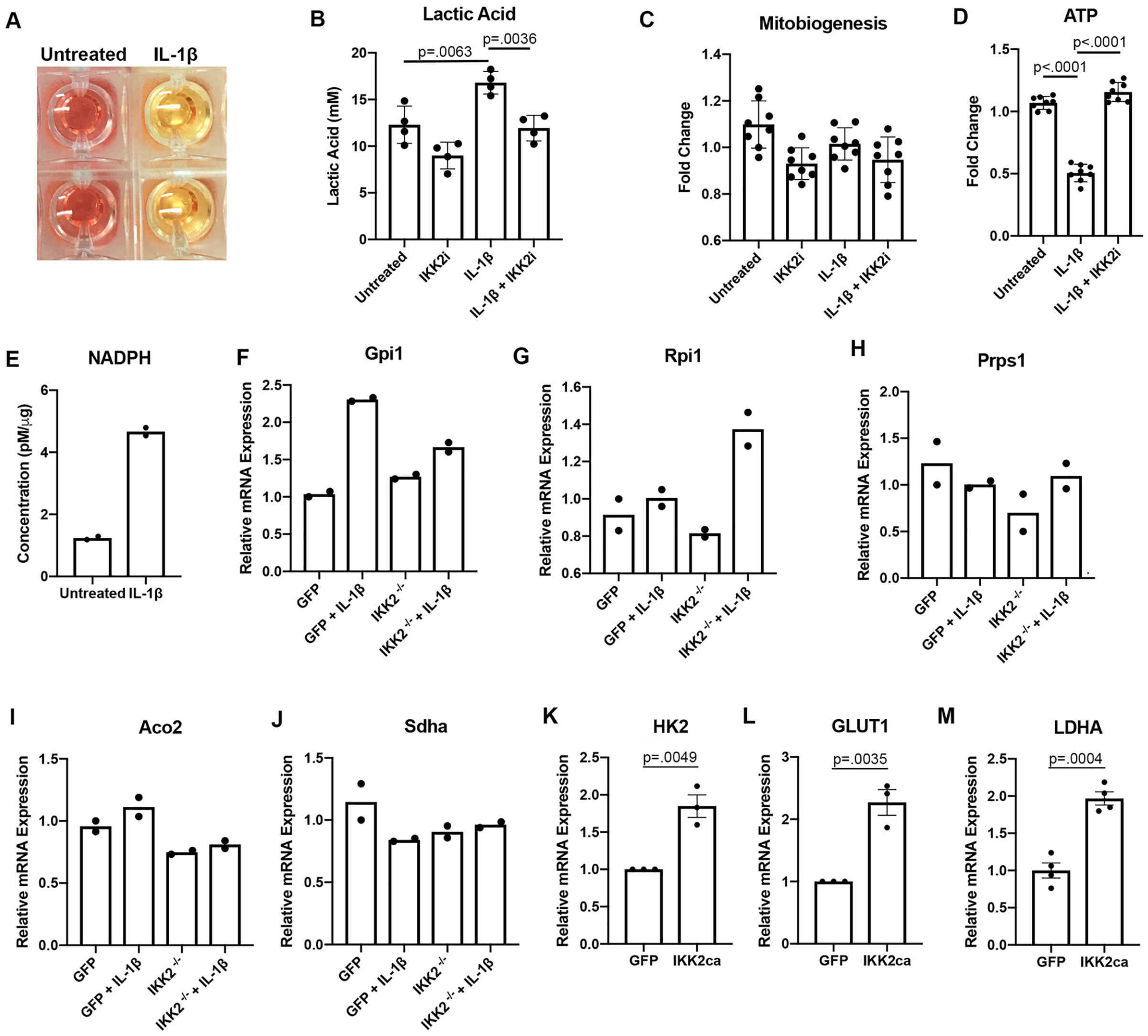
A
Untreated
IL-1β
B
Lactic Acid
Lactic Acid (mM)
p=.0063
p=.0036
Untreated
IKK2i
IL-1β
IL-1β + IKK2i
C
Mitobiogenesis
Fold Change
Untreated
IKK2i
IL-1β
IL-1β + IKK2i
D
ATP
Fold Change
p<.0001
p<.0001
Untreated
IL-1β
IL-1β + IKK2i
E
NADPH
Concentration (pM/μg)
Untreated
IL-1β
F
Gpi1
G
Rpi1
H
Prps1
Relative mRNA Expression
GFP
GFP + IL-1β
IKK2 -/-
IKK2 -/- + IL-1β
I
Aco2
J
Sdha
K
HK2
p=.0049
L
GLUT1
p=.0035
M
LDHA
p=.0004
GFP
IKK2ca

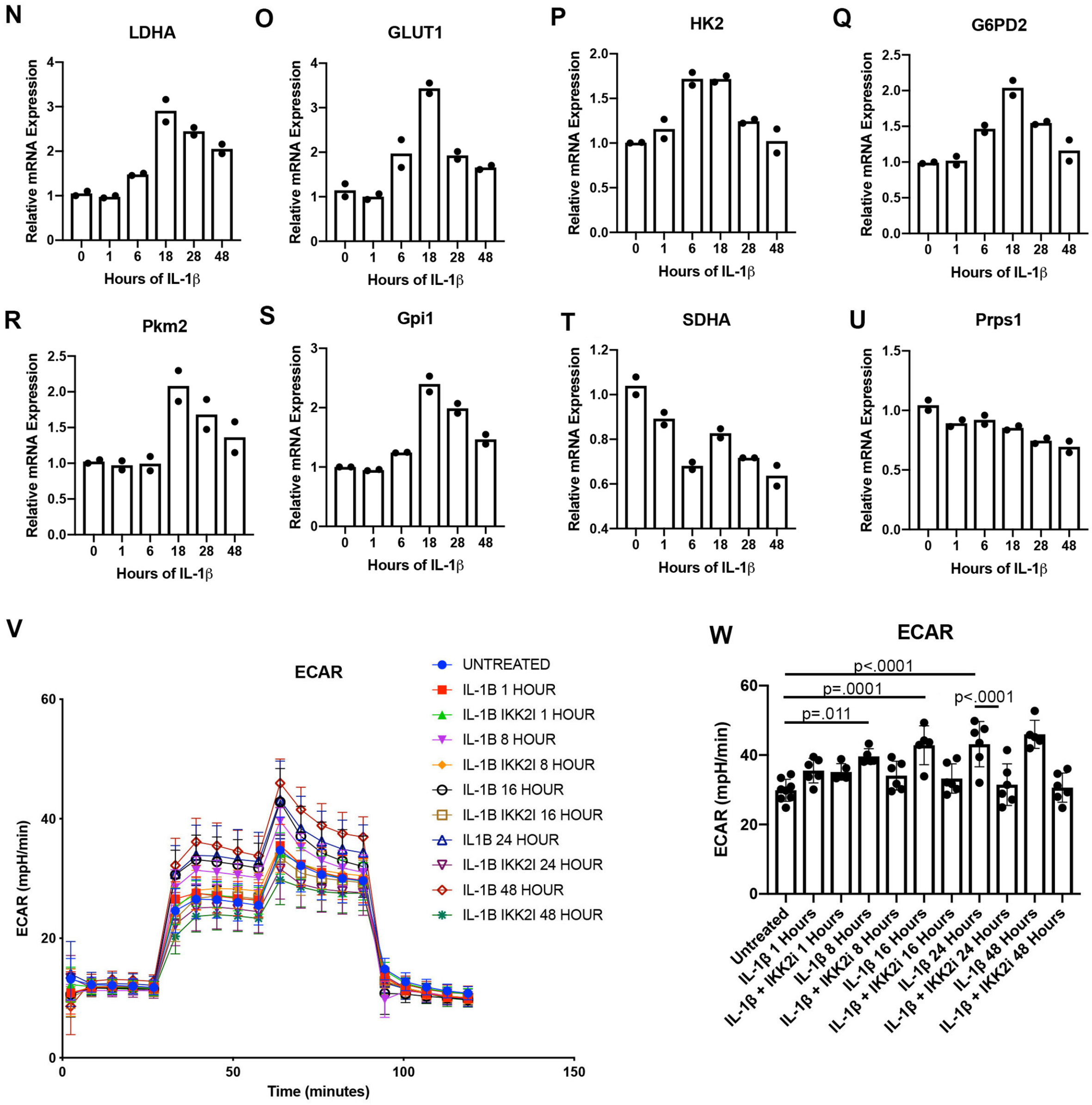

Supplementary Figure 1. Inflammation promotes aerobic glycolysis in chondrocytes (A) Representative image of chondrocytes cultured in phenol-red containing media in the presence or absence of IL-1β (10 ng/mL) for 48 hours displaying increased acidification. **(B)** Primary chondrocytes were treated with IL-1β and/or IKK2i (10 μM) for 24 hours. Lactic acid in chondrocyte supernatants was measured in biological replicates from independent experiments, with bars representing mean ± S.D for n=4 replicates. **(C)** Chondrocytes were treated for 24 hours. Biogenesis was measured by comparing the ratio of SDHA to Cox2 protein in cells, normalized to cell number. Graph

displays mean ± S.D. with n=8 replicates, representative of one of two experiments. **(D)** ATP levels were measured in chondrocytes treated with IL-1β and/or IKK2i for 48 hours. Data from two experiments, with n=4 samples each. Error bars represent mean ± S.D. of n=8 samples. **(E)** Primary chondrocytes were treated with IL-1β (10 ng/mL) for 24 hours. NADPH levels were measured and normalized to protein concentration, with bars representing mean of results from n=2 biologically independent replicates. **(F-J)** IKK2$^{f/f}$ chondrocytes were infected with adenoviral-GFP, or adenoviral-cre (labeled IKK2$^{-/-}$), then treated with IL-1β (10 ng/mL) for 24 hours. qPCR was performed for Gpi1, Rpi1, Prps1, Aco2, Sdha. Data shown is representative of one experiment out of three, with bar representing mean of technical duplicates. **(K-M)** Chondrocytes were retrovirally transduced with GFP or IKK2ca. 48 hours after transduction, HK2, GLUT1 and LDHA gene expression was measured. Bars represent mean ± S.E.M. from n=3 independent experiments. **(N-U)** Primary chondrocytes were treated with IL-1β (10 ng/mL) for various timepoints. Gene expression of LDHA, GLUT1, HK2, G6PD2, PKM2, Gpi1, SDHA, and PRPS1 was measured. Bars are mean of technical replicates, representative of one of two independent experiments. **(V)** Chondrocytes were treated for the indicated timepoints prior to performing seahorse assay. Measurements were performed every 5 minutes with bars representing mean ± S.D. for n=6 replicates for each condition, representative of one out of two independent experiments. **(W)** ECAR after oligomycin injection, with bars displaying mean ± S.D. from n=6 replicates. (K-M) Two-tailed student's T test. (B-D, W) One-way ANOVA followed by Tukey's multiple comparisons test. P values noted in figure.

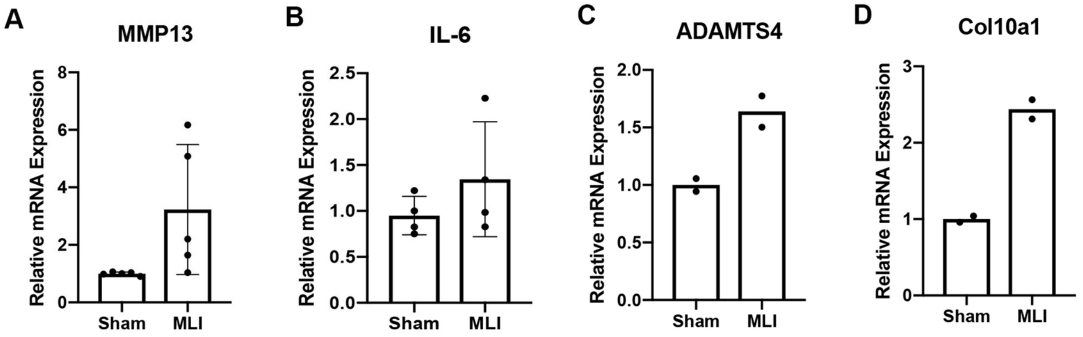

Supplementary Figure 2. MLI induces expression of catabolic and inflammatory mediators. (A-D) mRNA was isolated from articular cartilage of MLI compared to sham

joint 4 weeks after MLI surgery. Gene expression analysis for each mouse was performed by qPCR, with bars representing mean ± S.D for MMP13 and IL-6. Two-tailed student's T test was utilized for statistical analysis. (MMP13 (n=5 mice): P=.08, IL-6 (n=4 mice): P=.16, ADAMTS4 (n=2 mice), Col10a1 (n=2 mice))

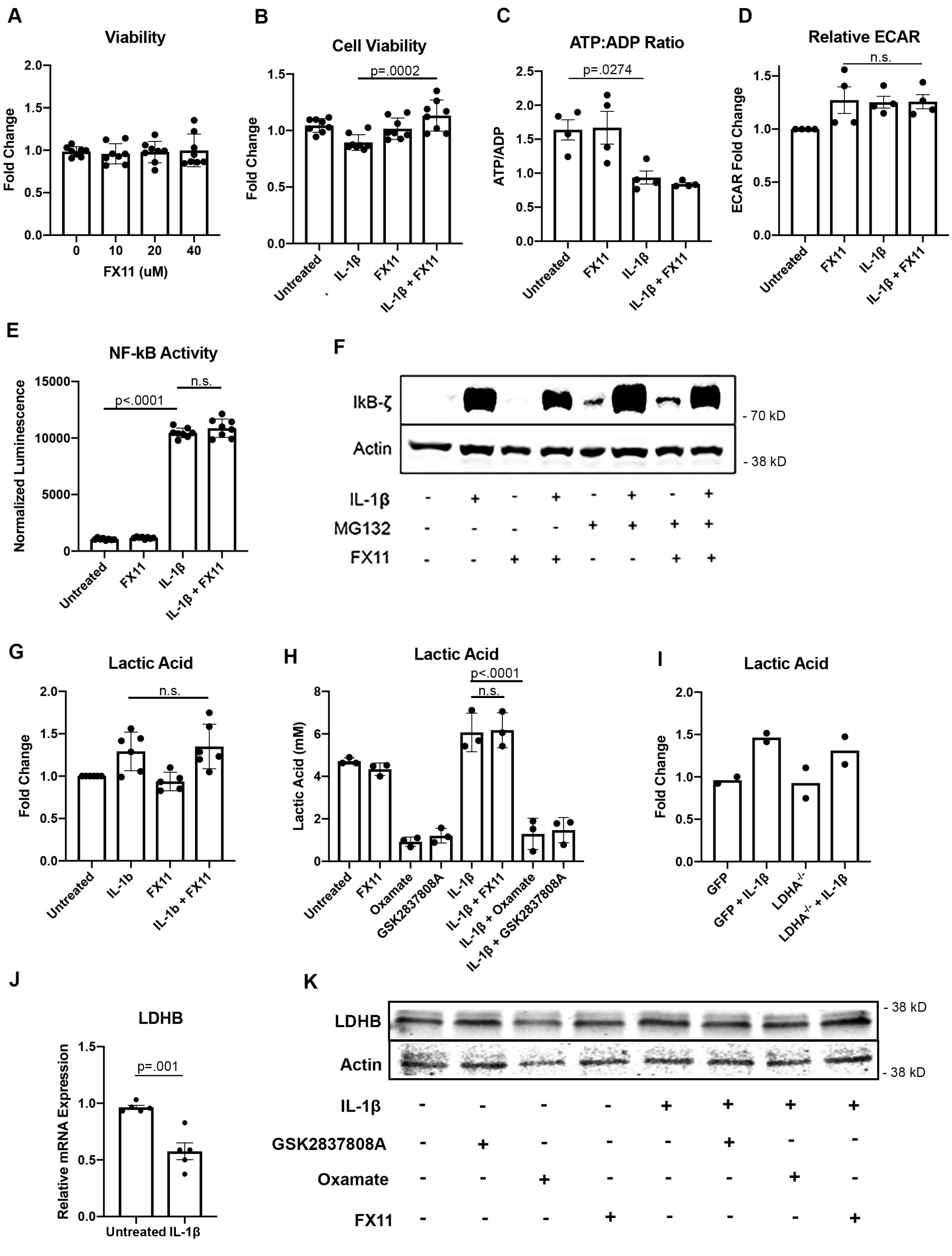
A
Viability
Fold Change
2.0
1.5
1.0
0.5
0.0
0
10
20
40
FX11 (uM)
B
Cell Viability
p=.0002
Fold Change
1.5
1.0
0.5
0.0
Untreated
IL-1β
FX11
IL-1β + FX11
C
ATP:ADP Ratio
p=.0274
ATP/ADP
2.5
2.0
1.5
1.0
0.5
0.0
Untreated
FX11
IL-1β
IL-1β + FX11
D
Relative ECAR
n.s.
ECAR Fold Change
1.5
1.0
0.5
0.0
Untreated
FX11
IL-1β
IL-1β + FX11
E
NF-kB Activity
p<.0001
n.s.
Normalized Luminescence
15000
10000
5000
0
Untreated
FX11
IL-1β
IL-1β + FX11
F
IkB-ζ
Actin
- 70 kD
- 38 kD
IL-1β - + - + - + - +
MG132 - - - - + + + +
FX11 - - + + - - + +
G
Lactic Acid
n.s.
Fold Change
2.0
1.5
1.0
0.5
0.0
Untreated
IL-1b
FX11
IL-1b + FX11
H
Lactic Acid
p<.0001
n.s.
Lactic Acid (mM)
8
6
4
2
0
Untreated
FX11
Oxamate
GSK2837808A
IL-1β
IL-1β + FX11
IL-1β + Oxamate
IL-1β + GSK2837808A
I
Lactic Acid
Fold Change
2.0
1.5
1.0
0.5
0.0
GFP
GFP + IL-1β
LDHA-/-
LDHA-/- + IL-1β
J
LDHB
p=.001
Relative mRNA Expression
1.5
1.0
0.5
0.0
Untreated
IL-1β
K
LDHB
Actin
- 38 kD
- 38 kD
IL-1β - - - - + + + +
GSK2837808A - + - - - + - -
Oxamate - - + - - - + -
FX11 - - - + - - - +

Supplementary Figure 3. LDHA inhibition regulates inflammation independent of lactate production. (A-B) MTT assay for viability was performed on chondrocytes treated with FX11 in the presence or absence of IL-1β for 24 hours. Bars represent mean ± S.D. for n=6 replicates. **(C)** Chondrocytes were treated with IL-1β (10 ng/mL) in the presence or absence of FX11 (40 µM) for 24 hours before ATP:ADP ratio was measured. Error bars represent mean ± S.E.M. from n=4 biologically independent replicates. **(D)** Sternal chondrocytes were treated with IL-1β (10 ng/mL) and/or FX11 (40 µM) for 48-72 hours prior to performing seahorse assay. ECAR was measured 10 minutes after start of assay. Error bars represent mean ± S.E.M. from n=4 independent experiments. **(E)** Chondrocytes were isolated from NF-κB-Luciferase reporter mice. Luciferase measurement was performed on chondrocytes treated with IL-1β (10 ng/mL) in the presence or absence of FX11 (40 µM) for 24 hours, normalized to protein. Data shown is mean ± S.D. for n=8 replicates. **(F)** Primary chondrocytes were treated with IL-1β (10 ng/mL) for 24 hours in the presence or absence of FX11 (40 µM) prior to 6 hour MG132 treatment. Immunoblotting was performed for IkB-ζ. **(G)** Chondrocytes were treated with IL-1β (10 ng/mL) in the presence or absence of FX11 (40 µM) for 24 hours. Supernatant lactic acid was measured. Bars represent mean ± S.E.M. for n=6 independent experiments. **(H)** Chondrocytes were treated with IL-1β (10 ng/mL) in the presence of GSK2837808A (10 µM), oxamate (10 mM) or FX11 (40 µM) for 24 hours for supernatant lactic acid measurement. Bars represent mean ± S.D. from n=3 biological replicates. **(I)** $LDHA^{f/f}$ chondrocytes were infected with adeno-GFP or adeno-cre, then treated with IL-1β (10 ng/mL) for 24 hours. Supernatant lactic acid was measured. Bars are mean of technical duplicates. **(J)** Chondrocytes were treated with IL-1β (10 ng/mL) for 24 hours. LDHB gene expression was measured, with bars displaying mean ± S.E.M. from n=5 independent experiments. **(K)** Immunoblotting was performed for LDHB on samples treated with same conditions as in S3H. (A-E, G-I) One-way ANOVA followed by Tukey's multiple comparisons test. (J) Two-tailed student's T test. P values are noted in figure.

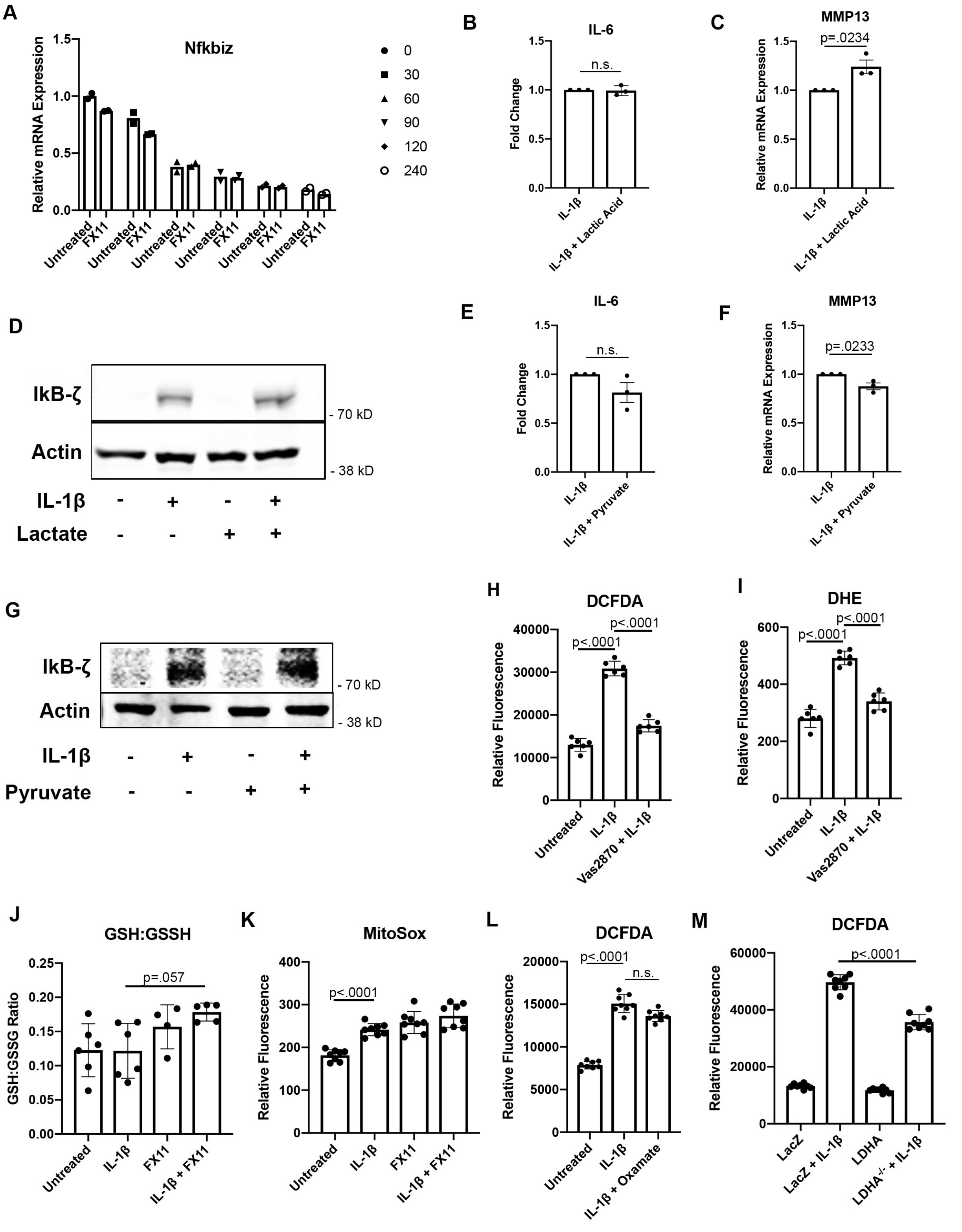

Supplementary Figure 4. LDHA interaction with NADH propagates ROS. (A) Chondrocytes were treated with IL-1β (10 ng/mL) in the presence or absence of FX11 (10

μM) for 24 hours, then treated with actinomycin D (6.5 ug/mL) for timepoints upto 4 hours. Gene expression of *Nfkbiz* was measured, with bars representing mean of technical duplicates. **(B-C)** Chondrocytes were treated with IL-1β (10 ng/mL) and/or lactic acid (500 μM) for 24 hours. Gene expression of IL-6 and MMP13 was measured normalized to IL-1β treatment with bars displaying mean ± S.E.M. from n=3 independent experiments. **(D)** Immunoblotting was performed under the same conditions for IkB-ζ, representative of one of three independent experiments. **(E-F)** Primary chondrocytes were treated with IL-1β (10 ng/mL) and/or pyruvate (10 mM) for 24 hours. Gene expression of IL-6 and MMP13 was measured, with bars displaying mean ± S.E.M. from n=3 independent experiments. **(G)** Immunoblotting was performed for IκB-ζ protein. **(H-I)** Chondrocytes were treated with IL-1β in the presence or absence of Vas2870 (50 μM) for 24 hours. DCFDA or DHE was utilized to measure ROS levels. Error bars represent mean ± S.D. of n=8 replicates. **(J)** Primary chondrocytes were treated with IL-1β (10 ng/mL) for 24 hours in the presence or absence of FX11 (40 μM). GSH and GSSH were measured biological replicates across 2 independent experiments. Bars display mean ± S.D. for n=6. **(K)** Chondrocytes were treated with IL-1β (10 ng/mL) and/or FX11 (40 μM) for 24 hours. Mitochondrial ROS was measured using Mitosox. Error bars display mean ± S.D. for n=8 replicates. **(L)** Primary chondrocytes were treated with IL-1β (10 ng/mL) in the presence or absence of sodium oxamate (5 mM) for 24 hours. DCFDA assay was performed to measure intracellular ROS levels. Results represent one of three independent experiments, with error bars indicating mean ± S.D. for n=8 replicates. **(M)** *Ldha*$^{f/f}$ chondrocytes were infected with adeno-cre (LDHA$^{-/-}$) or adeno-LacZ, followed by IL-1β treatment for 24 hours. DCFDA assay was performed with bars representing mean ± S.D. from n=8 replicates, representing one of three independent experiments. (B-C, E-F) Two-tailed student's T test. (H-M) One-way ANOVA followed by Tukey's multiple comparisons test. P values are noted in figure.

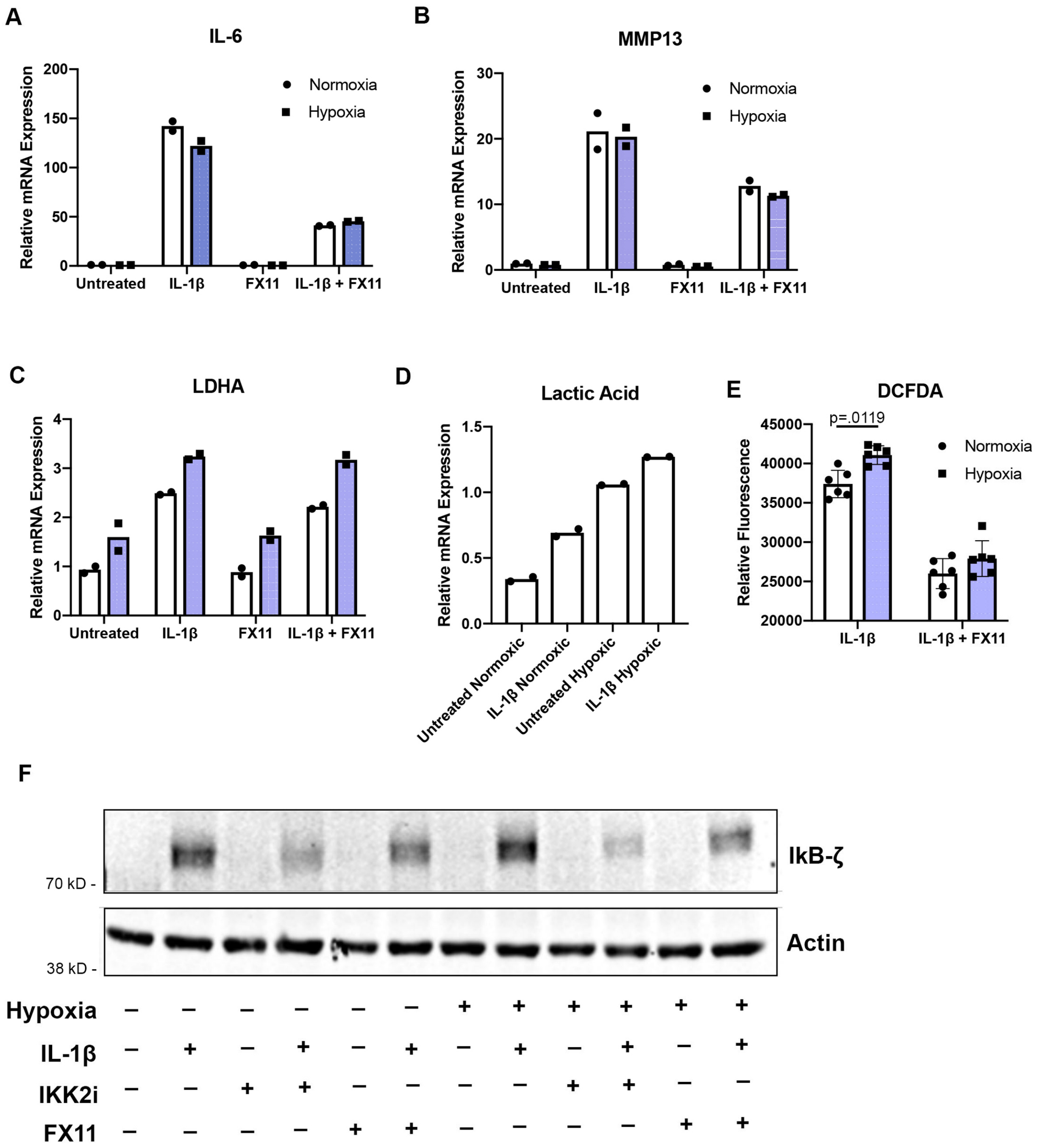

Supplementary Figure 5. Chondrocytes under hypoxia behave similar to normoxic conditions. (A) Chondrocytes were isolated from newborn pups and cultured in either 21% O_2 normoxic or 5% O_2 hypoxic conditions side by side. Cells were treated with IL-1β (10 ng/mL) in the presence or absence of FX11 (40 µM) for 24 hours. **(A-C)** Gene expression of IL-6, MMP13 and LDHA were measured. Bars represent mean of two technical replicates, with data representative of one out of two independent experiments.

(D) Lactic acid levels in the supernatant were measured in technical duplicates, with bars representing mean. **(E)** Cellular ROS levels were measured using DCFDA fluorescent probe under the same conditions. Bars represent mean ± S.D for n=6 replicates. Two-way ANOVA followed by Sidak's multiple comparisons test was utilized for statistical analysis. P values noted in figure. **(F)** Representative Western blot for IκB-ζ and actin from chondrocyte lysates treated as indicated under normoxic or hypoxic conditions, representative of one out of two independent experiments.

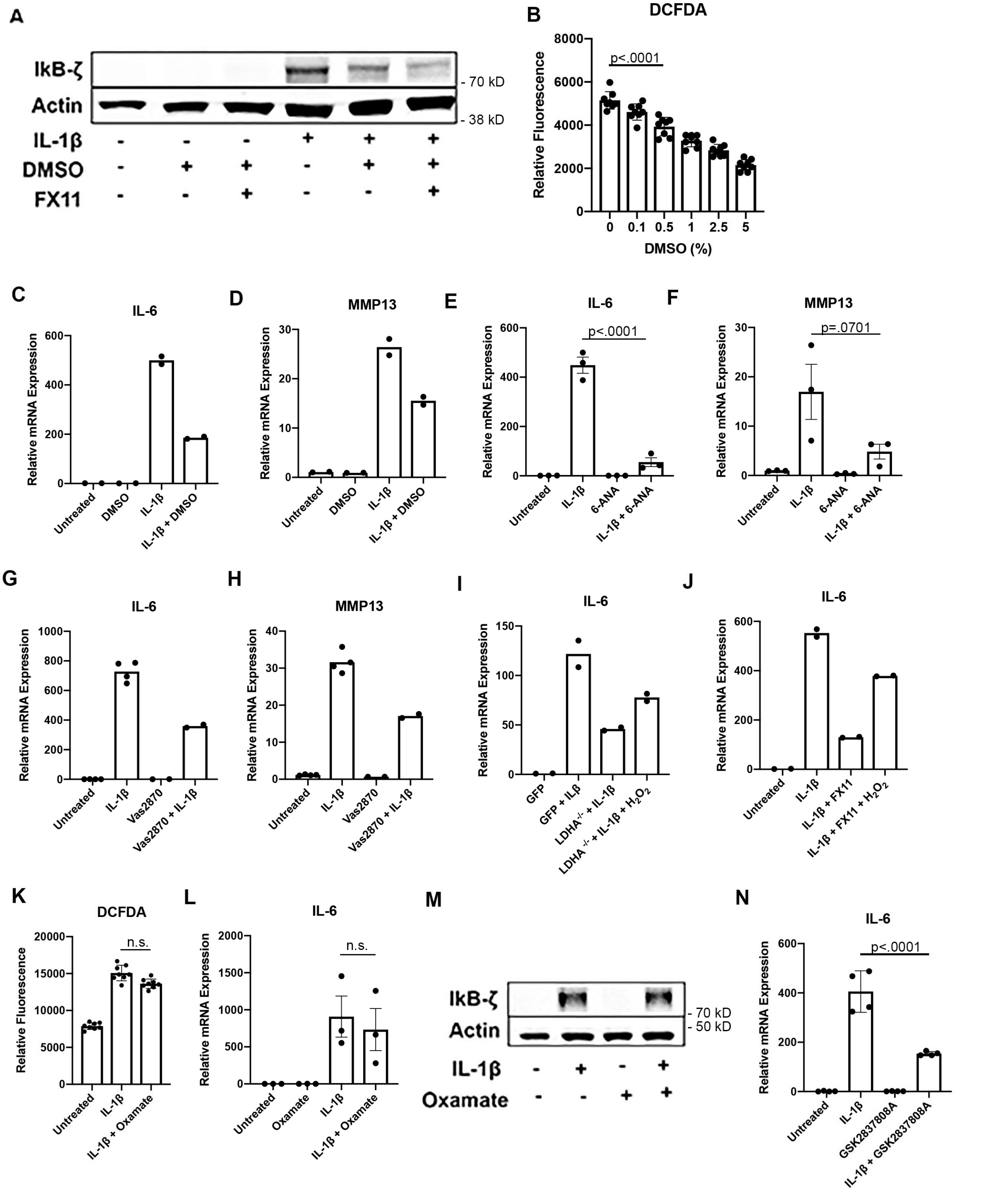

Supplementary Figure 6. ROS regulates stability of IκB-ζ protein. (A) Chondrocytes were treated with IL-1β and/or DMSO (1%) and FX11 (40 μM) for 24 hours. Western blotting was performed for IκB-ζ. **(B)** Chondrocytes were treated with increasing

concentrations of DMSO for 24 hours. Intracellular ROS was measured by DCFDA assay. Bars represent mean ± S.D. for n=8, representative of one out of two independent experiments. **(C-D)** Gene expression was measured on samples treated with IL-1β (10 ng/mL) and/or DMSO (1%). Bars are mean of technical duplicates from one representative experiment out of two. **(E-F)** Primary chondrocytes were treated with IL-1β (10 ng/mL) and/or 6-ANA (100 µM) for 24 hours. Gene expression was measured by qPCR. Bars are mean ± S.E.M. from n=3 independent experiments. **(G-H)** Primary chondrocytes were treated with IL-1β (10 ng/mL) in the presence or absence of Vas2870 (50 µM) for 24 hours. Gene expression of IL-6 and MMP13 was measured with bars representing mean of technical replicates, representative of one of three independent experiments. **(I)** WT and $LDHA^{-/-}$ chondrocytes were treated with IL-1β and H_2O_2 (50 µM) for 24 hours. Gene expression of IL-6 was measured. Graph displays data from one representative experiment out of three, with bar representing mean of technical replicates. **(J)** Primary chondrocytes were treated with IL-1β (10 ng/mL) in the presence or absence of FX11 (40 µM) and/or H_2O_2 (50 µM) for 24 hours. Gene expression of IL-6 was measured. Bar represents mean of technical replicates. **(K)** Primary chondrocytes were treated with IL-1β (10 ng/mL) and/or sodium oxamate (5 mM) for 24 hours. Intracellular ROS was measured by DCFDA assay, with bars representing mean ± S.D. for n=8 replicates. **(L)** Gene expression of IL-6 was measured, graph displays mean ± S.E.M. for n=3 independent experiments. **(M)** Western blotting was performed for IκB-ζ under these same conditions. **(N)** Primary chondrocytes were treated with IL-1β (10 ng/mL) in the presence or absence of GSK2837808A (10 µM) for 24 hours. Gene expression of IL-6 was measured by qPCR for n=4 replicates. Bars represent mean ± S.D. for n= 4 replicates. (B,E-F,K, L, N) One-way ANOVA followed by Tukey's multiple comparisons test. P values noted in figure.

Supplementary Table 1: qPCR primer list

Gene Name	Primer Sequence
Actin (Mouse)	F- 5'-CTAAGGCCAACCGTGAAAAG-3'
	R- 5'-ACCAGAGGCATACAGGGACA-3'
IL-6 (Mouse)	F- 5'-GAGGATACCACTCCCAACAGACC-3'

	R- 5’-AAGTGCATCATCGTTGTTCATACA-3’
MMP13 (Mouse)	F- 5’-GCCAGAACTTCCCAACCAT-3’
	R- 5’-TCAGAGCCCAGAATTTTCTCC-3’
IL-6 (Human)	F- 5’-CCAGCTATGAACTCCTTCTC-3’
	R- 5’-GCTTGTTCCTCACATCTCTC-3’
MMP13 (Human)	F- 5’-AATATCTGAACTGGGTCTTCCAAAA-3’
	R- 5’-CAGACCTGGTTTCCTGAGAACAG-3’
LDHA	F- 5’-GGATGAGCTTGCCCTTGTTGA-3’
	R- 5’-GACCAGCTTGGAGTTCGCAGTTA-3’
Glut1	F-5’-TCTCTGTCGGCCTCTTTGTT-3’
	R- 5’-GCAGAAGGGCAACAGGATAC-3’
IKK2	F- 5’-CCGGAAAGTGTCAGCTGTATC-3’
	R- 5’-CCTCAGCTGGAAGAAGGAGA-3’
G6pd2	F- 5’-CTGAATGAACGCAAAGCTGA-3’
	R- 5’-CAATCTTGTGCAGCAGTGGT-3’
Gpi	F- 5’-GTGGTCAGCCATTGGACTTT-3’
	R- 5’-CTTTCCGTTGGACTCCATGT-3’
Rpia	F- 5’-TGCAGCGAATAGCTGAAAGA-3’
	R- 5’-ACAGCCATTCGAAGTTCCAC-3’
Prps1	F- 5’-TTGATATCCCGGTGGACAAT-3’
	R- 5’-AGGGCCAGAAAAGATTCCAT-3’
Aco2	F- 5’-AATGGATGTACTCGTTGGGC-3’
	R- 5’-ACAGCCTACTGGTGACTCGG-3’
SDHA	F- 5’-AACACTGGAGGAAGCACACC-3’
	R- 5’-AGTAGGAGCGGATAGCAGGA-3’
NFKBIZ	F- 5’-TCTCACTTCGTGACATCACC-3’
	R- 5’-GGTTGGTATTTCTGAGGTGGAG-3’
NOX2	F- 5’-CCCTTTGGTACAGCCAGTGAAGAT-3’
	R- 5’-CAATCCCGGCTCCCACTAACATCA-3’
NOX4	F- 5’-GGATCACAGAAGGTCCCTAGCAG-3’
	R- 5’-GCGGCTACATGCACACCTGAGAA-3’
MCP-1	F- 5’-CATCCACGTGTTGGCTCA-3’
	R- 5’-GATCATCTTGCTGGTGAATGAGT-3’
ADAMTS4	F- 5’-CTTCCTGGACAATGGTTATGG-3’
	R- 5’-GAAAAGTCGCTGGTAGATGGA-3’
LDHB	F- 5’-AGTCTCCCGTGCATCCTCAA-3’

	R- 5’-AGGGTGTCCGCACTCTTCCT-3’
Actin (Human)	F-5’-CTAACCGCGAGAAGATGA-3’
	R-5’-CCAGAGGCGTACAGGGATAG-3’
NFKBIZ (Human)	F-5’-CCGTTTCCCTGAACACAGTT-3’
	R-5’-AGAAAAGACCTGCCCTCCAT-3’
G6PD2 (Human)	F-5’-AAGAACGTGAAGCTCCCTGA-3’
	R-5’-AATATAGGGGATGGGCTTGG-3’
