# Supplementary material for: LDHA-mediated ROS generation in chondrocytes is a potential therapeutic target for osteoarthritis
Source: Nat Commun. 2020 Jul 9;11:3427. doi: 10.1038/s41467-020-17242-0 (PMC7347613; doi:10.1038/s41467-020-17242-0)
Supplement: Supplementary file 3 — Description of Additional Supplementary Files [file 41467_2020_17242_MOESM3_ESM.pdf]

Description of Additional Supplementary Files

Supplementary Data 1

Description: Differentially Expressed Genes from RNA Sequencing. Related to Figure 1. All differentially expressed genes from the RNAseq dataset that had a P-value less than or equal to 0.05
