# Supplementary material for: LDHA-mediated ROS generation in chondrocytes is a potential therapeutic target for osteoarthritis
Source: Nat Commun. 2020 Jul 9;11:3427. doi: 10.1038/s41467-020-17242-0 (PMC7347613; doi:10.1038/s41467-020-17242-0)
Supplement: Supplementary file 5 — Reporting Summary [file 41467_2020_17242_MOESM5_ESM.pdf]

nature research

Corresponding author(s): Yousef Abu-Amer

Last updated by author(s): 6/15/2020

Reporting Summary

Nature Research wishes to improve the reproducibility of the work that we publish. This form provides structure for consistency and transparency in reporting. For further information on Nature Research policies, see Authors & Referees and the Editorial Policy Checklist .

Statistics

For all statistical analyses, confirm that the following items are present in the figure legend, table legend, main text, or Methods section.

n/a	Confirmed	
☐	☒	The exact sample size (*n*) for each experimental group/condition, given as a discrete number and unit of measurement
☐	☒	A statement on whether measurements were taken from distinct samples or whether the same sample was measured repeatedly
☐	☒	The statistical test(s) used AND whether they are one- or two-sided<br>*Only common tests should be described solely by name; describe more complex techniques in the Methods section.*
☒	☐	A description of all covariates tested
☒	☐	A description of any assumptions or corrections, such as tests of normality and adjustment for multiple comparisons
☐	☒	A full description of the statistical parameters including central tendency (e.g. means) or other basic estimates (e.g. regression coefficient) AND variation (e.g. standard deviation) or associated estimates of uncertainty (e.g. confidence intervals)
☐	☒	For null hypothesis testing, the test statistic (e.g. *F*, *t*, *r*) with confidence intervals, effect sizes, degrees of freedom and *P* value noted<br>*Give P values as exact values whenever suitable.*
☒	☐	For Bayesian analysis, information on the choice of priors and Markov chain Monte Carlo settings
☒	☐	For hierarchical and complex designs, identification of the appropriate level for tests and full reporting of outcomes
☒	☐	Estimates of effect sizes (e.g. Cohen's *d*, Pearson's *r*), indicating how they were calculated

Our web collection on statistics for biologists contains articles on many of the points above.

Software and code

Policy information about availability of computer code

Data collection	BioTek Gen5 used for microplate based assays such as DCFDA and MTT, Odyssey Image Studio V5 used for scanning fluorescent western blot membranes, Biorad Cfx manager used for collecting qPCR data, Seahorse Wave Version 13 used for collecting Seahorse data, Metamorph Image Analysis Software used for capturing images from microscope camera
Data analysis	Graphpad Prism 7.0 used to graph data and perform statistical analysis, Wave Version 13 used to generate Seahorse graphs, ImageJ V1.51 used for western blot images, Illumina bcl2fastq V2.20 used for processing raw data from RNA sequencing, R/Bioconductor Packages: EdgeR, Limma, GAGE, Pathview were combined for analysis of processed RNA sequencing data, KEGG database used for pathway analysis of RNA sequencing data

For manuscripts utilizing custom algorithms or software that are central to the research but not yet described in published literature, software must be made available to editors/reviewers. We strongly encourage code deposition in a community repository (e.g. GitHub). See the Nature Research guidelines for submitting code & software for further information.

Data

Policy information about availability of data

All manuscripts must include a data availability statement. This statement should provide the following information, where applicable:
- Accession codes, unique identifiers, or web links for publicly available datasets
- A list of figures that have associated raw data
- A description of any restrictions on data availability

Source data and raw images is provided for figures 1C-L, 2A, 3A-F,3I-M, 4A-L, 5A-E, 6A-G, 7B, 8A-F, 8I-J, S1B-W, S2A-D, S3A-K, S4A-M, S5A-F, S6A-N as a source data file. Additional data that support the findings of this study are available from the corresponding author upon reasonable request, including supporting data for representative experiments. All mice and material are subject to appropriate material transfer agreement. All raw and processed files for RNA-sequencing can be found on the GEO repository (Accession number: GSE148159, https://www.ncbi.nlm.nih.gov/geo/query/acc.cgi?acc=GSE148159)

Field-specific reporting

Please select the one below that is the best fit for your research. If you are not sure, read the appropriate sections before making your selection.

- [x] Life sciences
- [ ] Behavioural & social sciences
- [ ] Ecological, evolutionary & environmental sciences

For a reference copy of the document with all sections, see nature.com/documents/nr-reporting-summary-flat.pdf

Life sciences study design

All studies must disclose on these points even when the disclosure is negative.

Sample size

Sample size calculations were not performed prior to starting study. Sample sizes were deemed to be sufficient based on size of effects seen and reaching statistical significance based on the available samples. For in vivo BLI experiments, n=4 was used and experiments were repeated twice. For experiments in which large biological difference was expected such as OARSI scoring of cartilage rescue from MLI-induced OA by LDHA , at least n=3 was utilized and experiments were repeated twice. For human data, at least n=12 was used for gene expression experiments due to large variability in human samples.

Data exclusions

It was predetermined that data would only be excluded if there was an issue with sample quality, such as poor mRNA quality or if they fell outside 1.5 x IQR of the first or third quartile, a well accepted method for identifying outliers. Outliers were excluded from Fig 1E, 1F and S4G. Otherwise, data were included and displayed.

Replication

All experiments were performed in at least triplicate, unless otherwise stated with data showing similar trends between each experiment, especially if a representative experiment is performed. All experiments were successfully replicated with similar results, even if fold change values may be varied between biological samples.

Randomization

Randomization was performed when performing MLI surgeries on mice, with the surgeon unaware of the genotype of the mice at the time of surgery. All mice were fed tamoxifen chow to induce cre-recombination. Littermate mice were ultimately compared based on genotype.

Blinding

For histological analysis, slides were blinded for sectioning and scoring. Investigators were not blinded during sample collection for qPCR, western blotting experiments or DCFDA assays. However, these results of these assays are not subjective and provide quantitative data, removing the need for blinding. Only histological analysis required subjective grading of samples.

Reporting for specific materials, systems and methods

We require information from authors about some types of materials, experimental systems and methods used in many studies. Here, indicate whether each material, system or method listed is relevant to your study. If you are not sure if a list item applies to your research, read the appropriate section before selecting a response.

Materials & experimental systems

n/a	Involved in the study
[ ]	[x] Antibodies
[x]	[ ] Eukaryotic cell lines
[x]	[ ] Palaeontology
[ ]	[x] Animals and other organisms
[x]	[ ] Human research participants
[x]	[ ] Clinical data

Methods

n/a	Involved in the study
[x]	[ ] ChIP-seq
[x]	[ ] Flow cytometry
[x]	[ ] MRI-based neuroimaging

Antibodies

Antibodies used

anti-LDHA (Cat# PA5-27406, Invitrogen, 1:1000), anti-LDHB (Cat#14824-1-AP, Proteintech, 1:1000), anti-IκB-ζ (Cat# 14-16801-82, Invitrogen, 1:1000), anti-Actin (Cat# A228, Sigma, 1:5000), anti-p-IKK2 (ab5915, Abcam, Cambridge, UK 1:200), anti-MMP13 (Ab39012, Abcam, Cambridge, UK, 1:200), anti-IkB-ζ (NBP1-89835, Novus, Colorado, US. 1:200), anti-rabbit IRDye 680RD (926-68073, Licor, Lincoln, Nebraska, 1:10000), anti-mouse IRDye 800 CW (926-32212, Licor, Lincoln, Nebraska, 1:10000), anti-rat IRDye 800CW (926-32219, Licor, Lincoln, Nebraska, 1:10000), Anti-Rabbit IgG Biotinylated (BP-1100, Vector Labs, Burlingame, CA)

Validation

All antibodies were commercially validated for the application used. In addition, all antibodies used for western blotting showed bands at the expected sizes.

anti-LDHA (Cat# PA5-27406, Invitrogen) https://www.thermofisher.com/antibody/product/LDHA-Antibody-Polyclonal/PA5-27406
- "This antibody was verified by Independent antibody to ensure that the antibody binds to the antigen stated". Antibody was validated for western blotting by Thermo. It was also published in PMID 25032859.

anti-LDHB (Cat#14824-1-AP, Proteintech) https://www.ptglab.com/products/LDHB-Antibody-14824-1-AP.htm
- Product was validated for western blotting using mouse brain and heart tissue and published 15 times such as PMID 21325052

anti-IκB-ζ (Cat# 14-6801-82, Invitrogen) https://www.thermofisher.com/antibody/product/IkB-zeta-Antibody-clone-LK2NAP-Monoclonal/14-6801-82
- "This LK2NAP antibody has been tested by western blotting on reduced lysates at > 1 µg/mL"
- Published in PMID 29934320

anti-Actin (Cat# A228, Sigma) https://www.sigmaaldrich.com/catalog/product/sigma/a2228?lang=en&region=US
- Published 2582 times

anti-p-IKK2 (ab59195, Abcam, Cambridge, UK) https://www.abcam.com/ikk-beta-phospho-y199-antibody-ab59195.html
- Validated for IHC-P using human breast cancer tissue
- Published in mouse tissue in PMID 30322615

anti-MMP13 (Ab39012, Abcam, Cambridge, UK) https://www.abcam.com/mmp13-antibody-ab39012.html
- Tested for IHC-P by Abcam
- Published in PMID 30685700 in chondrocytes

anti-IkB-ζ (NBP1-89835, Novus, Colorado, US) https://www.novusbio.com/products/ikb-zeta-antibody_nbp1-89835
- Recommended by Novus for IHC-P, validated in human bone marrow and cerebrum

Biotinylated Anti-Rabbit IgG (BP-1100, Vector Labs, Burlingame, CA. https://vectorlabs.com/rtu-biotinylated-horse-anti-rabbit-igg-antibody.html#documents_
- "The biotinylated secondary antibodies are conjugated to ensure the maximum degree of labeling without compromising the specificity or affinity of the antibody"
- 25 citations have used this antibody

anti-mouse IRDye 800 CW (926-32212, Licor, Lincoln, Nebraska, 1:10000, https://www.licor.com/bio/reagents/irdye-800cw-donkey-anti-mouse-igg-secondary-antibody)
- Validated for western blotting

Anti-rat IRDye 800CW (926-32219, Licor, Lincoln, Nebraska, 1:10000, https://www.licor.com/bio/reagents/irdye-800cw-goat-anti-rat-igg-secondary-antibody)
- Validated for western blotting

Anti-Rabbit IgG Biotinylated (BP-1100, Vector Labs, Burlingame, CA, https://www.licor.com/bio/reagents/irdye-680rd-donkey-anti-rabbit-igg-secondary-antibody)
- Validated for western blotting

Animals and other organisms

Policy information about studies involving animals; ARRIVE guidelines recommended for reporting animal research

Laboratory animals	Male, C57BL6 mice were utilized for the experiments. Mice were aged 10 weeks before surgery was performed and sacrificed at various timepoints after surgery depending upon experimental outcomes. Aggrecan-ERT2-cre mice, IKK2ca flox/flox, LDHA flox/flox, Nfkbiz flox/flox mice were all bred on a C57BL/6 background. Mice were housed in barrier facility at 5 or less per cage at 24-26 degrees Celsius with humidity ranging between 30-60% with 12 hour light/dark cycles. For primary sternal chondrocyte isolation, mouse pups aged P1-P3 were utilized.
Wild animals	Study did not involve wild animals .
Field-collected samples	Field samples were not collected.
Ethics oversight	Washington University School of Medicine Institutional Animal Care and Use Committee (IACUC) approved all animal models utilized in this study.

Note that full information on the approval of the study protocol must also be provided in the manuscript.
